# Case Report: Atrial myxoma combined with hyperthyroidism in an adolescent with literature review

**DOI:** 10.3389/fonc.2023.1158385

**Published:** 2023-05-01

**Authors:** Mengjin Hao, Libo Wang, Dashi Ma, Weiying Guo

**Affiliations:** ^1^Department of Endocrinology and Metabolism, The First Hospital of Jilin University, Changchun, Jilin, China; ^2^Department of Pediatric Gastroenterology Unit, The First Hospital of Jilin University, Changchun, Jilin, China; ^3^Department of Cardiac Surgery, The First Hospital of Jilin University, Changchun, Jilin, China

**Keywords:** atrial myxoma, adolescent female, Graves’ disease, Carney Complex, diagnosis

## Abstract

The most common primary cardiac tumors in adults are atrial myxomas, with adolescent-onset being uncommon. In this case report, a 15-year-old female was hospitalized with cerebrovascular embolism and later diagnosed with a left atrial myxoma. She had previously shown signs of distal vascular micro thrombosis, including recurring bilateral lower extremity rash, which are crucial for the early diagnosis and differential diagnosis of atrial mucinous neoplasm. We reviewed the various clinical symptoms and diagnostic approaches to identify left atrial mucinous neoplasm. This patient also had a combination of endocrine-related diseases. We reviewed the diagnostic approach for the Carney Complex (CNC) and discussed the role of thyroid disease in diagnosing CNC.

## 1 Introduction

A primary cardiac tumor an atrial myxoma originates from primitive mesenchymal stem cells and can differentiate into cardiomyocytes, neuroendocrine cells, and endothelial cells. The exact mechanism of its formation is unknown (1). Atrial myxoma is extremely rare in adolescents, occurring at a rate of <5%; in adults, it is the most common primary cardiac tumor, accounting for approximately 50% of all cardiac tumors (2). Graves’ disease is the most common cause of hyperthyroidism and is more prevalent in women. In this case report, an adolescent female with Graves’ disease was diagnosed with an atrial myxoma following recurrent cerebral embolism.

## 2 Case report

**Figure 1:**
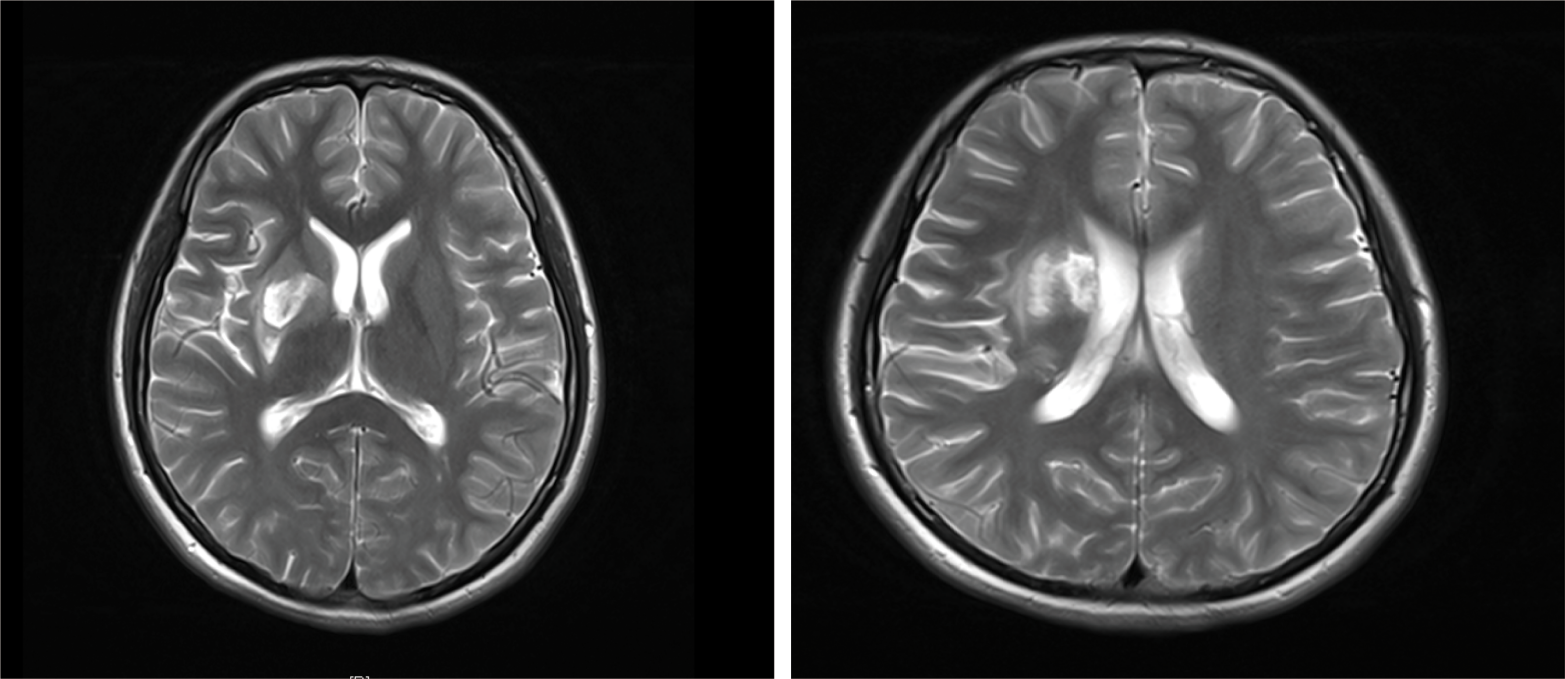
T2-weighted image of MRI: there are acute cerebral embolism lesions in multiple parts of the head.

**Figure 2:**
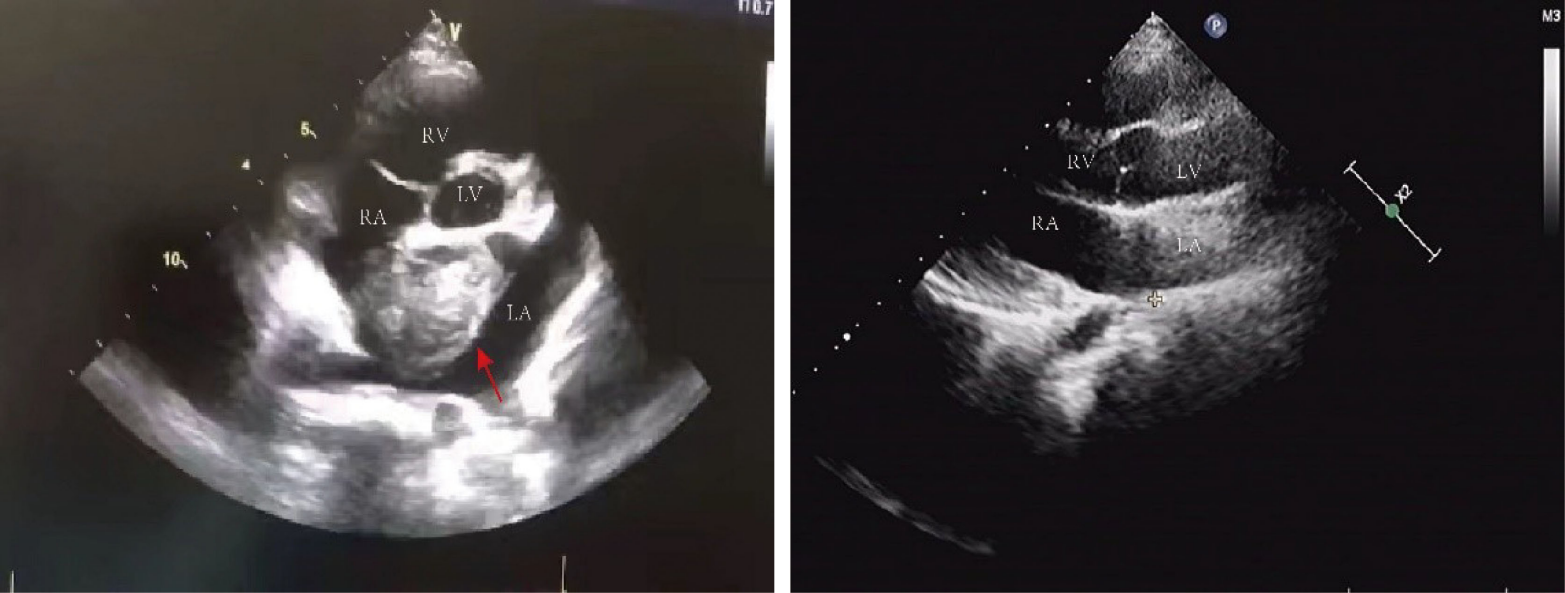
Echocardiography: there was a mass of 36 mm X 44 mm in size in the left atrium with roughly uniform echogenicity (red arrows pointing to the tumor in the left picture). The post-operative ultrasound is shown on the right picture. LV, left ventricle; RV, right ventricle; LA, left atrium; RA, right atrium.

A 15-year-old female adolescent visited the hospital following an emotional episode. She experienced a headache, a crooked mouth, and acute left limb immobility. The patient was examined for Graves’ disease at the age of 13 (May 2020) due to palpitations, wasting and irritability and has been treated with oral methimazole (at a current maintenance dose of 2.5 mg) during this period until now, with no family history of thyroid disease. Furthermore, she had a red rash on both her feet and pain several times before admission. There was no family history of drug usage or use of any other substances. Her blood pressure was 114/58 mmHg. Pulse was 86 beats/min, the patient was conscious, had proptosis (+), no rash, no pharyngeal congestion, no enlargement of the tonsils, no enlargement of the superficial lymph nodes, and no abnormalities were discovered on the cardiopulmonary and abdominal examinations. During a neurological examination, it was discovered that the corners of the mouth are skewed to the right, the left nasolabial fold becomes shallow, indicating facial nerve palsy, the muscle strength of the left upper limb was grade 4/5. The muscle strength of the left lower limb was grade 3/5 (using the Medical Research Council Manual Muscle Testing Scale for Assessing Muscle Strength). The left Babinski’s sign was positive. The patient’s neurological examination revealed all of the above-mentioned anomalies, which point to an upper motor neuron lesion and call for additional imaging tests to confirm the diagnosis. The biochemical results can be seen in **Table 1**. The cranial magnetic resonance imaging (MRI) (**Figure 1**) suggested multiple aberrant new signs in the brain, which were thought to be caused by ischemia due to the patient’s obvious neurological signs. Several high-coagulation risk factors and additional immunological markers were also ruled out. The echocardiography (**Figure 2**) revealed a mass in the left atrium that was generally hyperechoic and measured 36 mm × 44 mm. The electrocardiogram (ECG) indicated sinus rhythm, electric axis right deviation, and T-wave depression in II, III, aVF, and V4–V6 leads. The effective mitral regurgitant orifice area was 2.6 cm2 with mild regurgitation. The pedicle connects the tumor to the atrial septum, and the cardiac cycle affects the morphology of the tumor. During diastole, the tumor prolapsed through the mitral valve into the left ventricle, and during systole, it returned to the left atrium. It was also strongly suggestive of an atrial myxoma. The patient had an acute cerebral embolism, and the cardiac surgery department recommended elective surgery following acute phase (usually 3–4 weeks). Interestingly, this patient experienced a second transient ischemic attack 20 days following the initial cerebral embolism, with symptoms including aphasia and weakness in right limb that resolved independently after 30 minutes. This attack was discovered to be accompanied by a scattered light red rash on both feet that had macules and faded with pressure but did not protrude from the skin’s surface, accompanied by pain. The differential diagnosis of red rash includes infectious diseases (measles, rubella, scarlet fever, typhoid spotted rash, septicemia), immune system diseases (kawasaki disease, allergic purpura), hematologic diseases (infectious mononucleosis, thrombocytopenic purpura) and allergic reactive diseases (urticaria, eczema, drug rashes). It was determined to be secondary to either micro-embolic phenomena or vasospasm in combination with the rheumatology and dermatology consultation. The patient received a simple surgical resection to prevent the recurrence of cerebral embolism and other embolism sites. An open incision procedure was carried out for surgical treatment through the anterior median sternal incision. Intraoperative extracorporeal circulation was established. Intraoperative cross-clamping time was 28 minutes, while cardiopulmonary bypass time 43 minutes. The total blood loss was 300 ml, and 200 ml of blood was transfused. After surgery, the patient fully recovered without experiencing complications like bleeding or infection. Moreover, a histological examination confirmed the atrial myxoma diagnosis. There were no further manifestations of cerebral embolism or rash during the postoperative follow-up. The patient and family expressed great satisfaction with the treatment of this disease, as stated in the patient’s perspective at the end of the article.

**Table 1:** Biochemical data of thyroid function.

Item Name	Test Value	Normal Range
thyrotropin receptor antibody (A-TSHR) (IU/L)	4.640	0-1.75
thyroid-stimulating hormone (TSH) (uIU/mL)	1.86	1.35-4.94
free thyroxine 3 (FT3) (pmol/L)	3.37	2.43-6.01
free thyroxine 4 (FT4) (pmol/L)	12.59	9.01-19.05
thyroglobulin antibodies (A-Tg) (IU/mL)	>1000	0-4.11
thyroid peroxidase antibodies (A-TPO) (IU/mL)	616.2	0-5.61

## 3 Discussion

**Figure 3:**
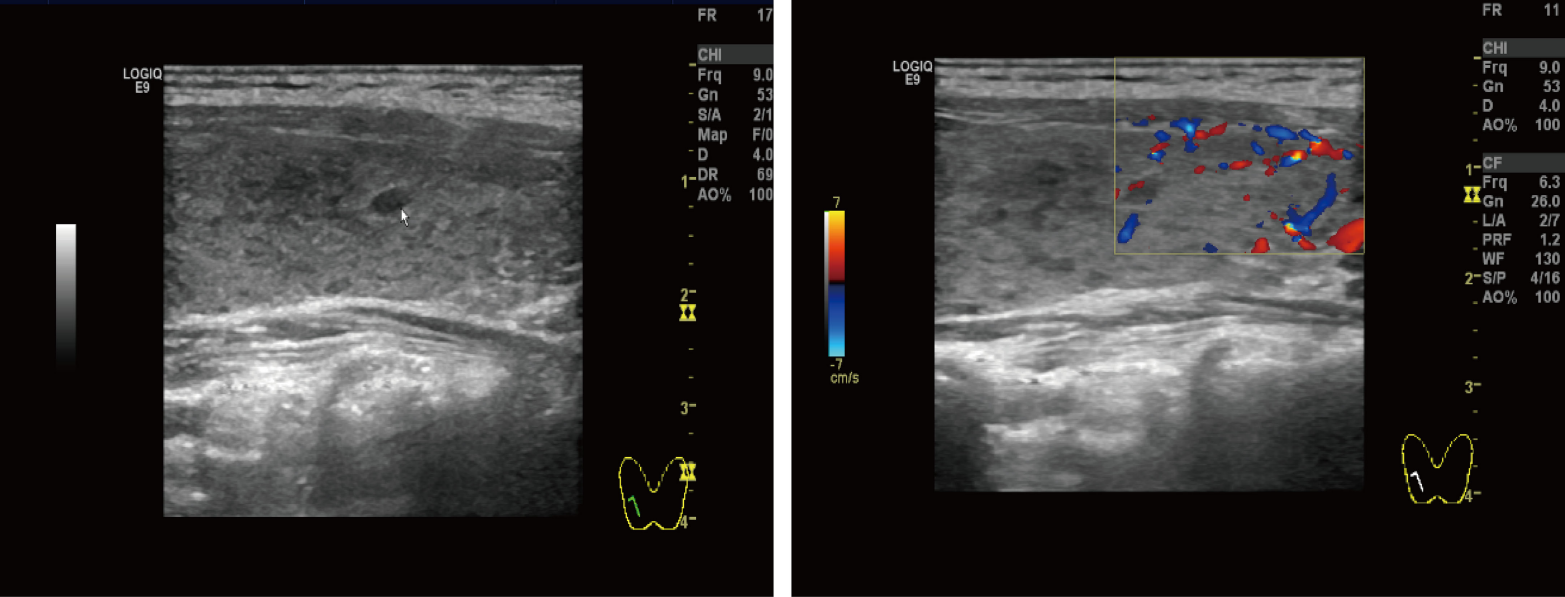
Thyroid ultrasound: Multiple nodules were present in both lobes of the thyroid gland. The larger nodule in the right lobe was located in the middle and lower part, measuring about 4.0 mm × 2.3 mm, with clear borders, regular shape, and substantial hypoechoic nodules. The larger nodule in the left lobe was located in the lower part, with a size of about 3.4 mm × 1.8 mm, with clear borders, regular shape, and substantial hypoechoic nodules.

The most common primary cardiac tumor in adults is atrial myxoma, which affects more women than men and is more common in the left atrium (60%–75%) than the right atrium (20%–28%). Ten percent of these tumors are familially inherited carney complex (3, 4). The size and location of the myxoma influence the clinical signs of left atrial myxoma. They include obstructive symptoms, embolic symptoms, and constitutional symptoms (1, 5). **Table 2** summarizes 11 cases of patients with left atrial mucinous tumors, four of which began in adolescence and the remaining seven in adulthood. Most patients also had systemic symptoms such as fever, malaise, slurred speech, limb immobility, and decreased muscle strength. A few patients presented with respiratory distress and rash. Two patients had hyperthyroidism, one of which was reported by Tack C et a.l (6), who discontinued postoperative anti-thyroid medication and did not have a recurrence of hyperthyroidism, which is different from our case. In most cases we reviewed, the atrial myxoma was detected by echocardiography as the initial examination. In one case, an atrial mucinous aneurysm was diagnosed based on pathological findings following embolization of the abdominal aorta, right renal artery (RRA), and superior mesenteric artery (SMA). Myxoma at the mitral valve orifice, which blocks the valve from opening and closing normally and increases pressure in the left ventricle and left atrium, causing congestive heart failure, is the primary cause of the majority of obstructive symptoms. The clinical symptoms include pulmonary edema, dyspnea, orthopnea, syncope, malaise, and palpitations. The fragility of the tumor, which results in bits coming loose and producing vascular embolisms in various parts of the body, is the primary source of embolic symptoms. The colloid type of myxoma frequently causes it (16). Together with cardiac manifestations, some extracardiac symptoms are more likely to appear clinically. The most common is embolism of the neurological vessels, which can manifest as a transient ischemic attack, hemiparesis, loss of vision, syncope, and seizures. Acute attacks frequently accompanied by a trigger of strenuous physical activity or emotional agitation characterize the presentation of cerebral embolism. Other arteries may be affected (renal artery, iliac artery, and peripheral vessels). Fever, anorexia, arthralgia, and weight loss are constitutional symptoms. This systemic symptom, similar to an inflammatory response, may be caused by the tumor-inducing the release of cytokines. Interleukin-6 has reportedly been linked to constitutional symptoms (17, 18). Echocardiography is essential when diagnosing atrial myxoma. The ultrasound presentation of a typical left atrial myxoma can be seen while the tumor morphology changes with the cardiac cycle (19). Additionally, transesophageal echocardiography, cardiac MRI, and computed tomography (CT) are available to support the diagnosis of atrial myxoma. The detection of atrial myxoma by echocardiography is superior to other methods because it enables dynamic visualization of tumor size, location, and mobility. MRI and CT scans are better equipped to evaluate mucinous tumor tissues and exclude tumor invasion of the heart wall because of their sensitivity to soft tissues (20, 21). In this case report, the patient had a bilateral lower extremity rash during the disease, in which distal vascular micro-thrombosis preceded the neurological event. The patient initially presented with recurrent cerebrovascular embolism as the first clinical manifestation. However, cardiac ultrasonography provided signs of the illness quite quickly. It had a higher propensity to lead to the misdiagnosis of other disorders, such as paraneoplastic syndrome, large vessel vasculitis, and allergic purpura (**Table 3**). There is evidence that patients with hyperthyroidism have an increased risk of atrial fibrillation, especially when risk factors such as excessive exercise, emotional stress, smoking, and alcohol consumption are present. Graves’ disease was the patient’s underlying disease, and paroxysmal atrial fibrillation cannot be ruled out as a possibility. Particularly, this patient had emotional agitation and early signs of cerebral embolism. A cerebral embolism may result from atrial fibrillation dislodging atrial myxoma fragments into the bloodstream. Besides myxoma embolisms, patients suffering from atrial fibrillation may experience also thromboembolism (generally due to the presence of a thrombus in the left atrial appendage). This patient also has a combination of Graves’ disease (endocrine disease) and should be alerted to Carney Complex (CNC). CNC is an autosomal dominant disorder that results in abnormal protein kinase A function due to mutations in the *PRKAR1A* gene. It is familial and affects multiple organ systems, presenting as atrial myxomas, skin nevi, spots and skin myxomas, and endocrine-related tumors. Atrial myxomas are one of them, and individuals with CNC are frequently younger when they acquire them (3, 22). The familial type of atrial mucinous tumor can be more accurately diagnosed and treated before malignant events such as embolism occur by being distinguished from disseminated myxoma. The diagnosis of CNC includes two main criteria (**Table 4**) that are imaging and histological confirmation or one of the main criteria combined with a PRKAR1A mutation or a first-degree relative with CNC (23). The thyroid ultrasound (**Figure 3**) showed no obvious tumor and no involvement of other systems, although the patient, in this case, had Graves’ disease and atrial myxomas. Thus, the diagnostic criteria of CNC were not met. Unfortunately, no clear conclusions were made about the relationship between hyperthyroidism and CNC. Approximately 77% of patients with CNC have skin pigmentation as their most prevalent clinical symptom, and 5% have thyroid nodules or thyroid tumors (24). Since 1997, Piper et al. have reported the production of concomitant hyperthyroidism in patients with CNC (25). Most patients with CNC had thyroid nodules and cystic lesions, making thyroid disease more common (26). As reported by Carney J et al., 26 patients with combined thyroid disease were examined, including 16 cases of follicular and nodular hyperplasia and 10 cases of follicular or papillary tumors, four of which were aggravated by hyperthyroidism. Therefore, hyperthyroidism should be considered one of the clinical manifestations of CNC (27). A systematic review of 20 studies that looked at thyroid hormone levels in patients with CNC found that subclinical hyperthyroidism was more common in patients with CNC than in the general population. These findings raise the possibility that subclinical hyperthyroidism is a phenotype of patients with CNC (28). Pringle D et al. previously discovered that thyroid knockout mice with the CNC pathogenic gene *PRKARLA* developed hyperthyroidism and thyroid cancer through animal studies (29). Therefore, different types of thyroid diseases in CNC should be noted and treated. The diagnosis in this case, where an adolescent female had a cerebral embolism and a recurring peripheral rash, should rule out atrial mucinous neoplasm and other diseases (**Table 3**). This report is distinctive for having a history of hyperthyroidism. Although there is no obvious connection between atrial myxoma and hyperthyroidism, long-term follow-up is necessary to differentiate diffused atrial mucinous neoplasm from CNC because the patient’s endocrine problem began in adolescence.

**Table 2:** Review of the clinical manifestations and laboratory in left atrial myxoma.

Reference	Age(years)/Gender	Clinical Symptoms	Laboratory Test	Comorbidities
Tack (6)	64/M	Weight loss, fatigue, fever	Eosinophilia, TSH<0.005μU/ml	hyperthyroidism
Cho (7)	49/F	fatigue, fever, transient ischemic attack, shortness of breath	C-reactive protein	None
Kumar (8)	65/F	dyspnea, fatigue	TSH<0.01mIU/L	hyperthyroidism hypertension
Macias (9)	13/F	fatigue, fever Rash	inflammatory markers	None
Bernatchez (10)	45/M	Extremity pain	None	None
Zhang (11)	42/M	Weakness of one limb	None	Obesity
Al-Mateen (12)	11/F;10/M	Limb immobility, slurred speech, visual disturbance, headache	ESR:40 mm/h	None
Kalra (13)	55/F	slurred speech, right arm weakness	None	None
Disney (14)	46/F	recurrent syncopal episodes	Thrombocytopenia	None
Schiele (15)	11/F	syncope, recurrent angina	NT -proBNP: 780 ng/L	Asthma

NT -proBNP, (N-terminal pro-brain natriuretic peptide); ESR, (Erythrocyte sedimentation rate); M, (Male); F, (Female).

**Table 3:** Differential diagnosis of left atrial myxoma.

Infectious	Intracardiac thrombus	Neoplastic causes	Autoimmunity
Infective endocarditis	Mitral-valve stenosis;	Metastatic tumors;	Vasculitis;
Septic emboli	Atrial fibrillation; Nonbacterial thrombotic Endocarditis;	Sarcoma; Lymphoma Myxoma; Hamartoma	Coagulopathy Allergic purpura

**Table 4:** Main criteria by imaging and histological examination for Carney Complex.

1. Spotty skin pigmentation with a typical distribution (lips, conjunctiva and inner or outer canthi, vaginal and penile mucosa)
2. Myxoma (cutaneous and mucosal) *
3. Cardiac myxoma*
4. Breast myxomatosis* or fat-suppressed MRI findings suggestive of this diagnosis
5. primary pigmented nodular adrenocortical disease (PPNAD)* or paradoxical positive response of urinary glucocorticosteroids to dexamethasone administration during Liddle’s test
6. Acromegaly due to GH-producing adenoma*
7. large cell calcifying Sertoli cell tumors (LCCSCT)* or characteristic calcification on testicular ultrasonography, in a young patient
8. Thyroid carcinoma* or multiple hypoechoic nodules on thyroid ultrasonography
9. Psammomatous melanotic schwannoma*
10. Blue nevus, epithelioid blue nevus (multiple)*
11. Breast ductal adenoma (multiple)*
12. Osteochondromyxoma*

*With histological confirmation.

## 4 Conclusion

The left atrial myxoma is a rare benign heart tumor, particularly if it develops in adolescents. Although cerebral embolism is a difficult cause to identify in children and delays the diagnosis of the disease, these clinical manifestations are typically associated with it. The patient also showed signs of microcirculatory thrombosis in both lower extremities (appearance of a rash in both lower extremities), which is clinically significant for the early diagnosis of atrial mucinous tumors prior to the disease progressing to cerebral embolism. The patient had several thyroid nodules and Graves’ and endocrine diseases. We considered whether it was related to CNC. Still, since the patient cannot be diagnosed with CNC since the current diagnostic criteria are not met, long-term follow-up observation of the patient is required. Surgery is the main treatment method, and several surgical procedures are used depending on the severity of the disease. Therefore, clinicians should identify left atrial myxoma in adolescents with recurrent rash and cerebral embolism to improve early diagnosis and treatment of such diseases and prevent more catastrophic consequences of massive cerebral embolism and myocardial infarction.

## Patient perspective

We were very fortunate to meet the doctors at the First Hospital of Jilin University, who helped my child find the cause of the brain embolism and successfully removed the lump. Now my child no longer has to worry about the sudden loss of body movement and speech, which has greatly improved our quality of life.

## Data availability statement

The original contributions presented in the study are included in the article/supplementary material. Further inquiries can be directed to the corresponding author.

## Ethics statement

The studies involving human participants were reviewed and approved by the Ethics Review Board of the First Hospital of Jilin University. The patients/participants provided their written informed consent to participate in this study. Written informed consent was obtained from the individual(s) for the publication of any potentially identifiable images or data included in this article.

## Author contributions

MH conceptualized and designed the case, wrote the initial manuscript, LW and DM collected pictures, carried out the initial analyses, WG reviewed and revised the manuscript. All authors contributed to the article and approved the submitted version.
